# Memory Is Enhanced at Behaviorally Relevant Times

**DOI:** 10.1371/journal.pbio.1000338

**Published:** 2010-03-16

**Authors:** Rachel Jones

**Affiliations:** Freelance Science Writer and Editor, Welwyn, Hertfordshire, United Kingdom

**Figure pbio-1000338-g001:**
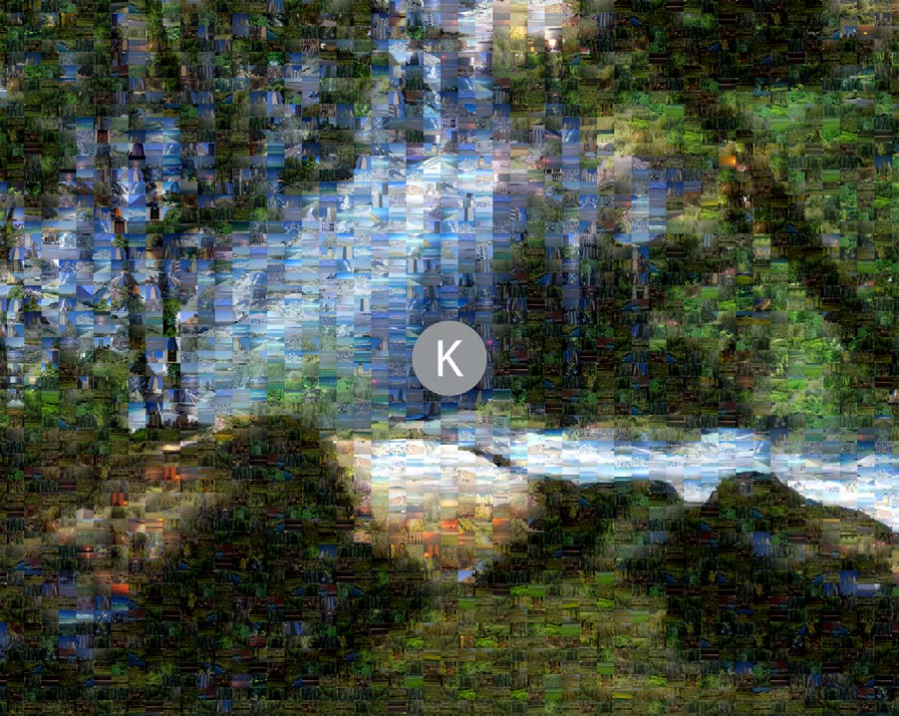
What determines whether an image is remembered or forgotten? New evidence suggests that attending to a task in the foreground could enhance memory for scenes in the background at behaviorally relevant points in time.

[Fig pbio-1000338-g001]Human memory for visual scenes varies according to a number of factors. These include the novelty of the scene, its behavioral relevance, and the subject's attention. Often, people will clearly remember details of the context in which they received shocking news—for example, remembering where they were and what they were doing when they heard that Elvis had died. This raises the possibility that another factor that can influence memory, even for irrelevant details, is the co-occurrence of a highly relevant stimulus.

In a new study in this issue of *PLoS Biology*, Lin et al. tested this theory using a technique called “rapid serial visual presentation” or RSVP. This protocol involves showing subjects a series of visual scenes, each scene being visible for only a short time, and then testing recognition memory by asking subjects whether a particular scene was present in the series.

The researchers began by presenting subjects with a series of 16 familiar scenes, and then testing whether they could identify which scenes had been presented. Surprisingly, performance on this task was no better than chance. However, when the series of scenes was presented at the same time as a letter recognition task to which the subjects had to pay attention, the subjects were significantly better at recognising scenes that had been presented simultaneously with the target letter (which was white among black distractor letters) than at recognising scenes that were presented with the distractors.

This supports the idea that a visual scene can be automatically committed to memory at Behaviorally relevant time points, such as when a target is presented during a task. But does this apply only to visual targets, or can other types of highly relevant stimulus also enhance visual scene memory? The authors addressed this question by presenting visual scenes while subjects attended to an auditory tone recognition task. As in the letter recognition task, subjects' memories were enhanced for the scene that was presented when the target tone was played.

To test whether the memory enhancement effect depended on the behavioral relevance of the target stimulus or on its physical novelty compared with the non-target stimuli, the authors repeated the letter recognition experiment, but instructed the subjects to ignore the letter stimuli and instead concentrate on the scene recognition task. Under these conditions, there was no memory enhancement; subjects were no more likely to correctly recognise the scene that had been presented with the white target letter than those that had been presented with the black distractor letters. This suggests that behavioral relevance, rather than perceptual novelty, is responsible for the enhanced scene memory in the preceding experiments.

These studies point towards a new mechanism by which attention can influence memory, in which memory for background scene can be enhanced even though attention is focused tightly on a target item in the foreground—or even on a target sound. Further studies are likely to investigate the parameters of this effect and to address the potential mechanisms of memory enhancement.

**Lin JY, Pype AD, Murray SO, Boynton GM (2010) Enhanced Memory for Scenes Presented at Behaviorally Relevant Points in Time. doi:10.1371/journal.pbio.1000337**

